# Physiological, transcriptomic, and genomic analysis unravels the response of Tatary buckwheat root to high ammonium stress

**DOI:** 10.3389/fpls.2025.1669365

**Published:** 2025-10-27

**Authors:** Changying Liu, Jiting Wang, Qingcheng Qiu, Daiying Xu, Xi Wu, Junjie Yin, Xiaoqin Zheng, Maoling Tan, Yan Wan, Wenjun Sun, Dabing Xiang

**Affiliations:** ^1^ Key Laboratory of Coarse Cereal Processing, Ministry of Agriculture and Rural Affairs, Sichuan Engineering and Technology Research Center of Coarse Cereal Industrialization, Chengdu University, Chengdu, Sichuan, China; ^2^ School of Food and Biological Engineering, Chengdu University, Chengdu, Sichuan, China

**Keywords:** genome re-sequencing, high NH_4_^+^, root, Tartary buckwheat, transcriptome

## Abstract

Toxicity caused by high ammonium severely affects plant growth and crop production, it is urgent to breed high NH_4_^+^-tolerant and high-yield plants. However, the molecular mechanisms on the response and tolerance of plant to high NH_4_^+^ remain poorly understood. In this study, four different genotypes of Tartary buckwheat (*Fagopyrum tataricum* Garetn.) were used to investigate the molecular mechanism on high NH_4_^+^ response by integrating physiological, transcriptome, and genome analysis. The root and shoot growth of Tartary buckwheat (TB) seedlings were significantly inhibited by 50 and 100 mmol/L NH_4_^+^ treatments. High NH_4_^+^ inhibits root growth by affecting activities of antioxidant enzymes, thereby suppressing plant growth. In total 426 high NH_4_^+^-responsive common differentially expressed genes (DEGs) were identified in TB. Most of DEGs involved in antioxidant enzyme system, hormone signaling, and N transport and assimilation were down-regulated by high NH_4_^+^. Co-expression analysis suggested the possible hub genes in regulating high NH_4_^+^ response, such as *FtNRT1.14*, *FtMYB61/52*, *FtbZIP6/34*, *FtNAC72/73*, and *FtLTP14.* 19 small secreted peptides (SSPs) encoding genes were respond to high NH_4_^+^, including *FtCLE7* and *FtCEP3*. The up-regulation of *FtCLE7* expression and down-regulation of *FtCEP3* expression may help plants to optimize root perception and response to high NH_4_^+^. Additionally, 443 genotype-specific high NH_4_^+^-responsive DEGs with sequence variation were identified by integrating transcriptome and genome re-sequencing data. The *TFs* such as *MYB*, *MADS*, and *LBD* genes and the *RLKs* such as *FtBAM1/3* may help TB to adapt to high NH_4_^+^. This work provides useful information for investigating the mechanisms on TB respond to high NH_4_^+^, and the candidate genes for breeding TB with high NH_4_^+^ tolerance were suggested.

## 1 Introduction

Nitrogen (N) is important for maintaining plant growth, development, and metabolism, which is also the major component of nucleic acids, proteins, chlorophyll, and many metabolites (Zhu et al., 2022b). N is taken up by plant roots in inorganic form such as nitrate, ammonium, and organic form including urea, amino acids, and peptides (Liu and von Wirén, 2017). Ammonium, nitrate, urea, and anhydrous ammonia were typically used as N fertilizers for improving crop growth and yield (Bittsánszky et al., 2015). Ammonium-related fertilizers are widely used due to ammonium is preferred N source for plants (Li et al., 2024). In practical crop production, urea is the worldwide leading N fertilizer, which is converted to ammonium by urease enzymes of soil microorganisms and then was absorbed by crops (Bittsánszky et al., 2015). However, ammonium-based fertilizers are excessively applied in intensive farming, which cause serious harm ammonium toxicity to plant growth and many environmental problems (Sun et al., 2022). The phenomenon of ammonium toxicity on plants was firstly found by Charles Darwin in 1882, which has now become a global problem (Bittsánszky et al., 2015). The main symptoms caused by ammonium toxicity include leaf chlorosis, rhizosphere acidification, decreases in root/shoot growth, cation uptake, photosynthesis, and yield, increases in photorespiration and oxidative stress, as well as osmotic and hormonal imbalance (Esteban et al., 2016). Therefore, it is urgent to lower the application of ammonium fertilizer and improve the tolerance and amelioration of crops against ammonium toxicity. Improving high ammonium tolerance and N use efficiency of crop through genetic engineering will be a promising approach for ensuring crop yield. However, the molecular mechanisms on the response and tolerance of plant to ammonium remain poorly understood. Elucidation of the molecular mechanism on the response to high ammonium could facilitate the improvement of high ammonium-tolerant and high-yield crops (Smoczynska et al., 2022).

In plants, ammonium was taken up by the roots via non-selective cation channels, potassium channels, aquaporins or ammonium transporters (AMTs) (Bittsánszky et al., 2015). Once ammonium enters into plant cells, it is assimilated into glutamine and glutamate via the glutamine synthetase (GS) and glutamate synthase (GOGAT) cycle. NADH-dependent glutamate dehydrogenase (GDH) synthesizes glutamate in the cytosol by using ammonium and 2-oxoglutarate (Liu and von Wirén, 2017). Besides, asparagine synthetase (ASN) and asparagine aminotransferase (ASP) can also transform ammonium into aspartate (Xu et al., 2012). Above foundational studies suggested that ammonium sensitivity of plant can be changed by manipulating AMT and N metabolism enzymes. Knockout of *AMT* genes in rice and *Arabidopsis* resulted in significant reduction in NH_4_^+^ uptake (Sonoda et al., 2003; Konishi and Ma, 2021). In poplar, overexpressing *GS1;2* gene increased tolerance to ammonium toxicity by maintaining carbon and N balance (Leng et al., 2024). In *Populus × xiaohei*, overexpression of alanine aminotransferase (*AlaAT*) gene enhances the tolerance of plants to ammonium toxicity (Yang et al., 2025). In addition, heterologous expression of a fungal *GDH* gene in rice alleviated ammonium toxicity and suppressed photorespiration (Yan et al., 2021).

Ammonium sensitivity of plants is a research hotspot, and the molecular regulatory mechanism of ammonium toxicity on plants was studied. It was found that hormone signaling is involved in high NH_4_^+^ response. The ethylene (ETH) precursor 1-aminocyclopropane-1-carboxylic acid (ACC) increases shoot sensitivity to NH_4_^+^, and ethylene-insensitive 3 (EIN3) leading to oxidative stress caused under NH_4_^+^ stress by inducing shoot ROS accumulation (Li et al., 2019). Abscisic acid (ABA) modulated ammonium stress response by regulating oxidative damage and NH_4_^+^ accumulation (Sun et al., 2020). Jasmonate meditated signaling also involves in regulating ammonium response by suppressing iron accumulation (Pandey et al., 2024). Some previous studies suggested that nitrate can alleviate ammonium toxicity in plants. In *Brassica napus*, nitrate alleviates ammonium toxicity of by balancing rhizosphere and intracellular pH, as well as accelerating NH_4_^+^ assimilation (Li et al., 2024). In *Arabidopsis*, nitrate transporter NRT1.1 and nitrate efflux channel SLAH3 form a functional unit to regulate nitrate-dependent ammonium toxicity alleviation and acidity tolerance (Zheng et al., 2015; Xiao et al., 2022). SNF1-related protein kinase 1 (SnRK1.1) participated in the nitrate-dependent ammonium toxicity alleviation by phosphorylating the C-terminal of SLAH3 (Sun et al., 2021a). Additionally, some transcription factors (TFs) are essential for the tolerance of plant to high ammonium. In *Arabidopsis*, *MYB28* and *MYB29* TFs play important roles in ammonium stress response, and mutation of both two genes lowered high ammonium tolerance (Coleto et al., 2021). *WRKY46* promotes ammonium tolerance by inhibiting ammonium efflux and repressing the expression of *NUDX9* and indole-3-acetic acid-conjugating genes (Di et al., 2021). *OsNLP3*-mediated nitrate signaling conferring rice with the ability to alleviate ammonium toxicity and to adapt to the high-ammonium condition (Yan et al., 2023). In barley (*Hordeum vulgare*), *HvMADS27* expression was significant decreased under ammonium stress condition, which regulates root architecture by modulating ABA metabolism (Smoczynska et al., 2022).

Although molecular mechanism on the response and tolerance to ammonium toxicity has been studied in model plants, very little information is available regarding non-model plants. Tartary buckwheat (*Fagopyrum tataricum* Gaertn.) is an important medicinal and edible crop with a worldwide distribution (Li et al., 2025). In this study, Tartary buckwheat (TB) was used to explore the physiological and molecular mechanisms of ammonium toxicity affecting the growth and development. In our previous studies, TB was used as a model plant to clarify the physiological mechanism of root in response to N, and the key genes involved in N uptake and utilization were revealed through genome re-sequencing and root transcriptome analysis (Liu et al., 2021a, 2021b, 2023a). However, the responsive mechanisms of TB in response to high NH_4_^+^ remain unclear. In TB cultivation, unreasonable application of N fertilizer, especially excessive application of ammonium-based fertilizers and urea, restricted the increase in yield of TB (Qiu et al., 2023). Therefore, the physiological mechanism of high NH_4_^+^ affecting the growth of four different TB varieties was analyzed in this study. The molecular mechanisms of root respond to high NH_4_^+^ were analyzed by transcriptome and genome analysis. The key genes regulating high ammonium response in TB root were suggested in this paper. This study will provide the basis for disclosing the molecular mechanism of TB and other crops under high NH_4_^+^ condition.

## 2 Materials and methods

### 2.1 Plant materials and treatments

Four TB varieties, Xiqiao No. 2 (XQ2), Fenghuang (FH), Yunqiao No. 1 (YQ1), and Yunqiao No. 2 (YQ2), were used in this study. These four varieties showed different response to N treatment (Qiu et al., 2023; Liu et al., 2023a). The seeds were sterilized with 0.7% sodium hypochlorite for 30 min and washed by ddH_2_O. And then, the seeds were placed in floating culture plates. Three days after seed germination, the seedlings with a root length of about 1.5 cm were selected and placed in Hoagland hydroponic solution containing different concentrations of NH_4_^+^ (1, 10, 50, and 100 mmol/L). (NH_4_)_2_SO_4_ was used as N source. The pH of the nutrient solution was adjusted to 5.8 ± 0.1. The nutrient solution was refreshed every three days. After six days of NH_4_^+^ treatment, the TB seedlings and roots were sampled, and the roots were stored at −80°C for subsequent experiments.

### 2.2 Morphology analysis of seedlings growth

The surface water of TB seedlings was dried with filter paper, and each plant was separated from the junction of roots and stems with scissors. The fresh weight of roots and shoots was measured, respectively. The total length of root and shoot, as well as lateral root number were measured by WinRHIZO scanning system (Version 2007d, Regent Instrument Inc., Canada).

### 2.3 Biochemical assays of hydroponic seedlings’ root

TB seedlings cultured in Hoagland nutrient solution containing 1 mmol/L and 100 mmol/L NH_4_^+^ for six days were collected. The roots were washed by ddH_2_O and were cut into pieces, which were used for determining the activities of superoxide dismutase (SOD), catalase (CAT), peroxidase (POD), and ascorbate peroxidase (APX), as well as malondialdehyde (MDA) content according to manufacturer’s instructions of test kits (Liu et al., 2022). All these kits were purchased from Nanjing Mofan Biotechnology Co., Ltd.

### 2.4 Transcriptomic analysis of TB root under high NH_4_^+^

Four varieties of TB seedlings cultured in nutrient solution of 1 mmol/L and 100 mmol/L NH_4_^+^ for six days were collected. The roots were washed with ddH_2_O, and then were cut off with sterilized scissors for transcriptome analysis. Weight the appropriate amount of frozen samples for total RNA extraction using Magnetic Tissue/Cell/Blood Total RNA Kit (Tiangen Biotech Co., Ltd., Beijing, China). The concentration and purity were detected by Thermo Scientific NanoDrop 2000 (Thermo Scientific, Waltham, Massachusetts, USA), and the integrity was detected by RNA 6000 Nano kit (Agilent Technologies Inc, California, USA). Total RNA with a total amount of ≥ 1 μg was used to construct the library using the NEBNext Ultra II RNA Library Prep Kit (New England Biolabs Inc., Massachusetts, USA). After that, Agilent 2100 Bioanalyzer (Agilent Technologies Inc, California, USA) and Agilent High Sensitivity DNA Kit (Agilent Technologies Inc, California, USA) were used to detect the quality of the library. The mixed library was gradually diluted and quantified, and then was sequenced on the Illumina sequencer by using PE150 mode. The raw sequencing data was submitted to NCBI Short Read Archive database (accession number: PRJNA1111253).

The raw data of the FASTQ were filtered to remove adaptor sequences at the 3’ end and the reads with an average mass fraction lower than Q20. The clean reads from each library were mapped to the reference genome of TB (Zhang et al., 2017; Liu et al., 2021a). Differentially expressed genes (DEGs) between two comparisons were analyzed using DESeq (v1.38.3) software. |log2FoldChange|>1 and significant *P*-value <0.05 was set as threshold to screen DEGs. Gene ontology (GO) enrichment analysis of DEGs was performed using topGO (v2.50.0). The DEGs were subjected to pathway analysis using KEGG database (http://www.genome.ad.jp/kegg/) (Liu et al., 2023a).

### 2.5 Quantitative RT-PCR verification

The SYBR Premix Ex Taq II (TaKaRa) kit was used for qRT-PCR analysis. The reaction system was as follows: 2 × TB Green Premix Ex Taq II Fast qPCR (12.5 μL), upstream primer (10 μM, 1 μL), downstream primer (10 μM, 1 μL), cDNA template (2 μL), and sterile water (8.5 μL). The reaction procedure was as follows: pre-denaturation at 95°C for 30 s, denaturation at 94°C for 5 s, annealing at 60°C for 10 s, extension at 72°C for 10 min, 40 cycles (Liu et al., 2021a). *FtActin7* was selected as the internal reference gene according to the transcriptome data, and each experiment was repeated three times. The gene expression was calculated by 2^-ΔΔCt^ method. The primers were showed in **Supplementary Table 1**.

### 2.6 Statistical analysis

All the data were processed by Excel 2019. IBM SPSS Statistics 25 software was used to analyze the significant differences between different samples by Duncan multi-factor and independent sample-T test. GraphPad Prism 9 and Adobe Illustrator CS6 were used for drawing pictures.

## 3 Results

### 3.1 High NH_4_^+^ seriously affects the growth of TB

**Figure 1:**
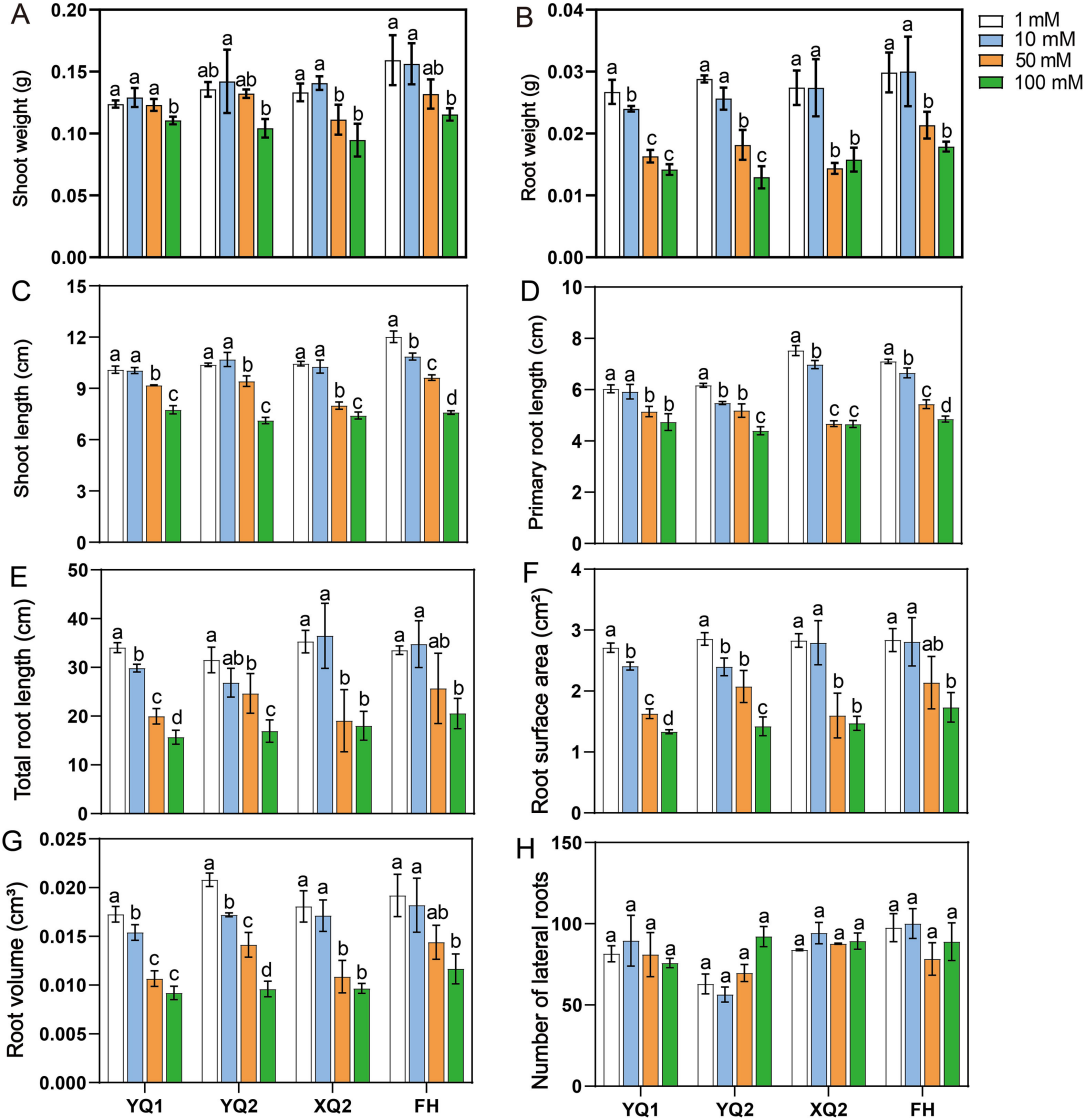
Effect of high NH_4_^+^ on the growth of TB hydroponics seedlings. **(A)** The weight of shoot under different concentrations of NH_4_^+^. **(B)** The weight of root under different concentrations of NH_4_^+^. **(C)** The length of shoot under different concentrations of NH_4_^+^. **(C, D)** The length of total root and primary root under different concentrations of NH_4_^+^. **(F-H)** The surface area and volume of root, as well as lateral root number of TB seedlings under high NH_4_^+^. Data are means ± SDs. The means were compared by Duncan’s test. Different treatments of each variety marked with different lowercase letters showed significant difference (*P* < 0.05).

As shown in **Supplementary Figure 1**, with the increase of NH_4_^+^ concentration, the development of hydroponic seedlings of four TB varieties was inhibited. Under high NH_4_^+^, the root and shoot weights of seedlings were significantly inhibited by 50 and 100 mmol/L NH_4_^+^ treatments (**Figures 1A, B**). At the same time, the hydroponic seedlings under high NH_4_^+^ tend to decrease the shoot length, primary root length, total root length, root surface area, and root volume, while the number of lateral roots did not change (**Figures 1C-H**). Therefore, it is speculated that high NH_4_^+^ may inhibit crop growth by affecting root development.

### 3.2 Biochemical analysis of hydroponics seedlings growth inhibition under high NH_4_^+^

**Figure 2:**
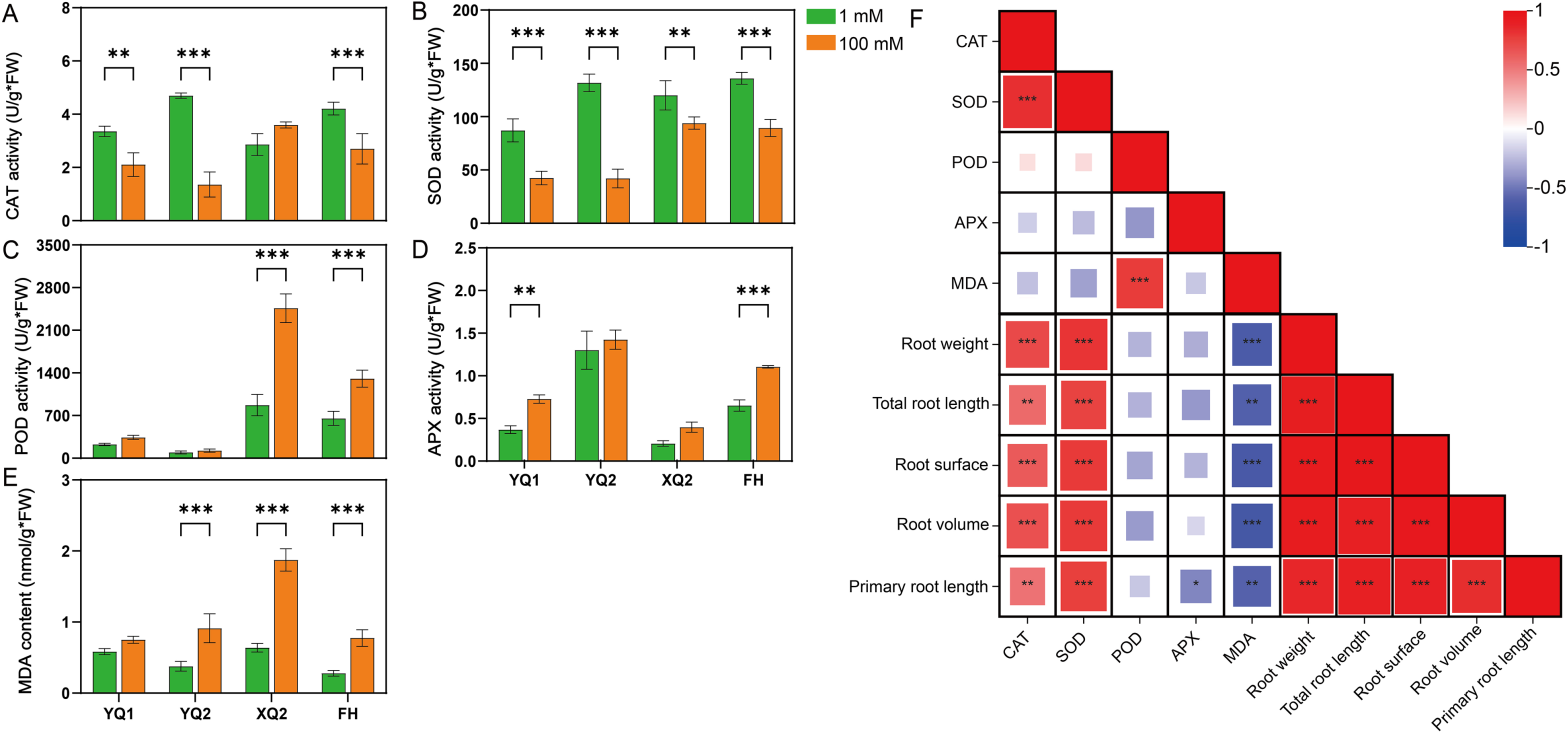
Stress analysis of TB hydroponics seedlings under high NH_4_^+^. **(A-E)** The activities of CAT, SOD, POD and APX, as well as MDA content in seedlings. **(F)** Correlation analysis between root morphology and enzyme activity of TB seedlings. Data are means ± SDs. The means were compared by Duncan’s test. ^∗^, ^∗∗^ and ^∗∗∗^ indicate *P* < 0.05, *P* < 0.01 and *P* < 0.001, respectively.

Biochemical analysis of the hydroponic seedlings’ roots under high NH_4_^+^ was conducted. Compared with NN (1 mmol/L NH_4_^+^) treatment, HN (100 mmol/L NH_4_^+^) significantly affects the MDA content and CAT, SOD, POD, and APX activities of TB seedling roots (**Figures 2A-E**). HN significantly increased the MDA content of seedling roots, indicating that roots were seriously damaged by high NH_4_^+^ (**Figure 2A**). The activities of CAT and SOD were inhibited, and the activities of POD and APX were increased (**Figures 2B-E**). Correlation analysis showed that MDA content was negatively correlated with root fresh weight, total root length, root surface area, root volume, and primary root length (*P* < 0.05) (**Figure 2F**). The activities of CAT and SOD were significantly positively correlated with root fresh weight, total root length, root surface area, root volume, and primary root length (*P* < 0.05) (**Figure 2F**).

### 3.3 The effect of high NH_4_^+^ on roots at transcriptome level

**Figure 3:**
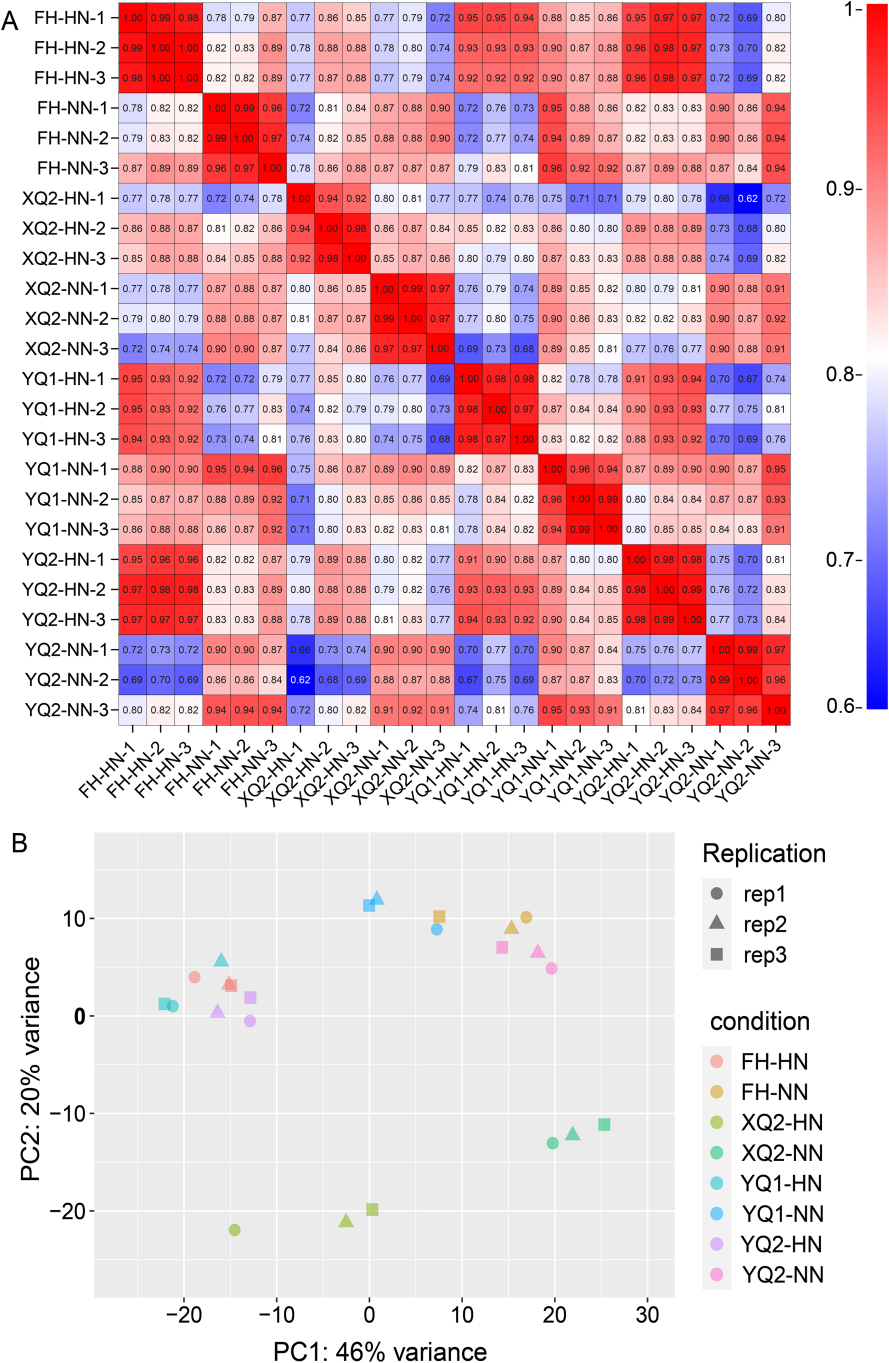
Correlation **(A)** and PCA **(B)** analysis of the RNA-seq data.

**Figure 4:**
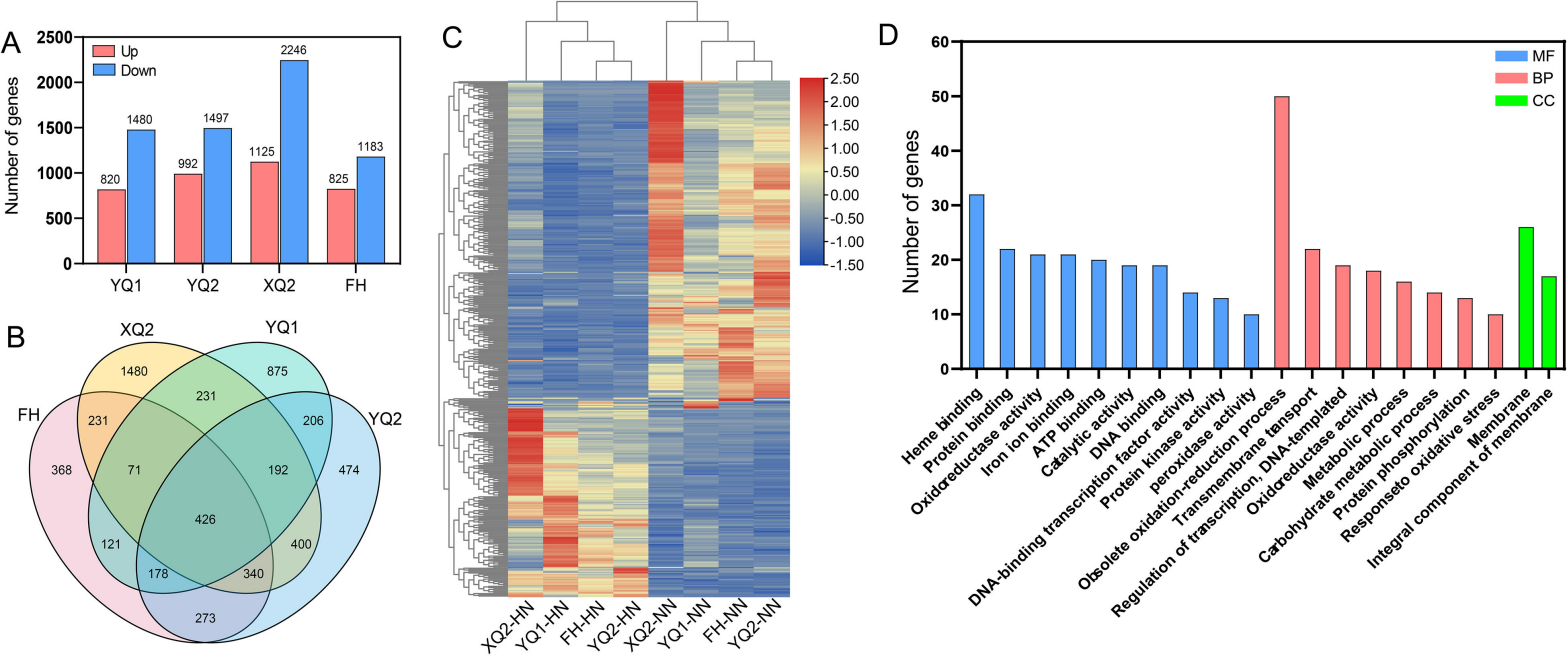
Analysis of DEGs in different TB varieties under high NH_4_^+^ treatment. **(A)** The DEGs number of each variety under high NH_4_^+^. **(B)** Venn diagram showing the overlapping DEGs among the four comparisons. **(C)** Heatmap of 426 common DEGs. **(D)** Gene ontology enrichment analysis of the 426 common DEGs.

In the present study, a transcriptome profiling analysis was performed to investigate the response of TB roots to high NH_4_^+^. A total of 152.13 Gb of high-quality bases were obtained from 24 samples (**Supplementary Table 2**). The proportion of bases whose quality values reached Q20 in each sample was greater than 97.25%, and proportion of bases whose quality values reached Q30 was greater than 92.38% (**Supplementary Table 2**). The expression data of all the detected TB genes were showed in **Supplementary Table 3**. It was found that the Pearson correlation coefficient between the three replicates of each treatment was greater than 0.9, and the samples had good repeatability (**Figure 3A**). Through PCA analysis, it was found that the samples of YQ1, YQ2, XQ2, and FH were significantly separated between NN and HN treatments (**Figure 3B**). A total of 2, 008 high NH_4_^+^-induced DEGs were screened in FH, including 825 up-regulated and 1, 183 down-regulated genes. XQ2 had 3, 371 DEGs, including 1, 125 up-regulated genes and 2, 246 down-regulated genes. YQ1 had 2, 300 DEGs, including 820 up-regulated and 1, 480 down-regulated genes. YQ2 had 2, 489 DEGs, including 992 up-regulated and 1, 497 down-regulated genes (**Figure 4A**). 12 genes, including *FtNRT1.10/1.22, FtNRT2.1/2.4*, *FtNRT3.1/3.2*, *FtNIR*, *FtGDH3*, *FtWRKY41*, *FtMYB52*, *FtLBD38*, and *FtNF-YA10*, were screened to verify the accuracy of transcriptome data by qRT-PCR. The expression levels of these genes determined by qRT-PCR were consistent with the expression patterns of RNA-seq data (**Supplementary Figure 2**).

426 common DEGs were found in all four varieties by Venn diagram analysis (**Figure 4B**; **Supplementary Table 4**). The results of the heatmap showed these genes have different expression patterns (**Figure 4C**). Among them, 153 genes were specifically up-regulated by high NH_4_^+^, 261 genes were specifically down-regulated, and 12 genes had different high NH_4_^+^-responsive patterns among the four TB varieties. GO enrichment analysis of 426 common DEGs showed that they were distributed in 200 GO categories. These genes were more enriched into peroxidase activity, heme binding, protein kinase activity, DNA-binding transcription factor activity, DNA binding, regulation of transcription, DNA-templated, metabolic process, transmembrane transport, protein phosphorylation, response to oxidative, obsolete oxidation-reduction process, membrane, and integral component of membrane (**Figure 4D**).

### 3.4 Expression analysis of the common DEGs in response to high NH_4_^+^

**Figure 5:**
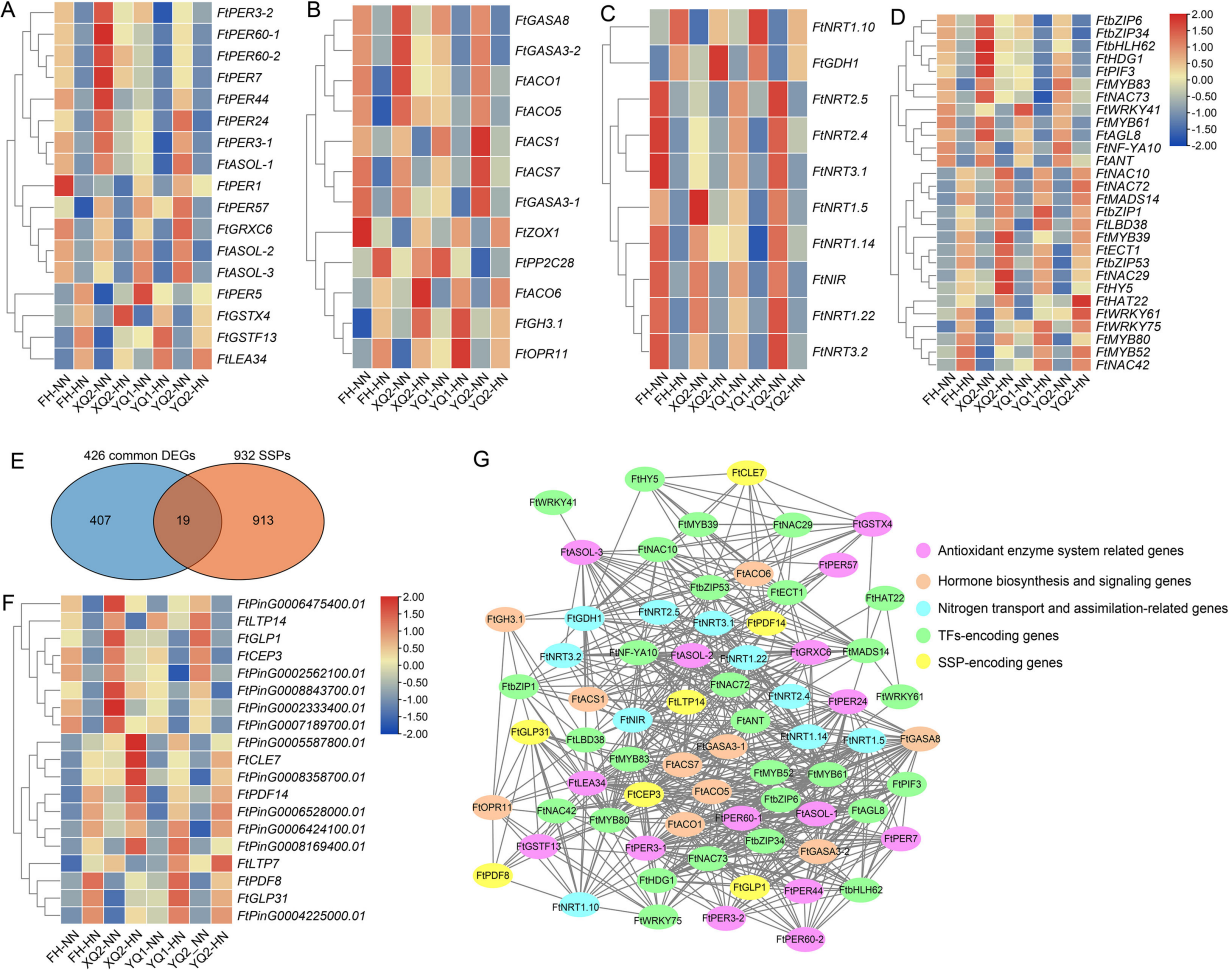
Identification of the key high NH_4_^+^-responsive genes from the 426 high NH_4_^+^-responsive DEGs. **(A-D)** Expression analysis of the genes involved in antioxidant enzyme system, hormone signaling and nitrogen transport, as well as transcription factors encoding genes. **(E)** Venn diagram showing the overlapping genes between 426 high NH_4_^+^-responsive DEGs and 932 *SSP* genes. **(F)** Expression analysis of the 19 DEGs encoding SSPs. Color scale on the heatmaps indicates the degree of expression: red, high expression; green, low expression. **(G)** The correlations among the key high NH_4_^+^-responsive DEGs. The pearson’s correlation coefficient was applied for correlation analysis. The correlation index >0.8 or < − 0.8 was set as the cut-off. The results were performed by Cytoscape software.

17 DEGs involved in antioxidant enzyme system were found in the 426 common NH_4_^+^-responsive DEGs (**Supplementary Table 5**). Among the ten *PER* genes, the expression of nine genes was significantly inhibited by HN treatment. The expression of *FtPER5* (FtPinG0000858000.01) was significantly inhibited by HN treatment in YQ1, while it was up-regulated in YQ2, XQ2, and FH (**Figure 5A**). The expression of three *ASOL* and one *GRX* was significantly inhibited by HN treatment, while two *GST* and one *LEA* genes were up-regulated by HN treatment (**Figure 5A**).

12 genes involved in hormone biosynthesis and signaling were found (**Supplementary Table 5**). Four ETH pathway genes were significantly inhibited by HN treatment, while the expression of *FtACO6* (FtPinG0005322400.01) showed the opposite expression pattern (**Figure 5B**). The three GA signaling genes were significantly inhibited by HN treatment. The genes involved in auxin (IAA) and jasmonic acid (JA) biosynthesis were up-regulated by HN treatment. The expression patterns of genes involved in cytokinin (CTK) and ABA biosynthesis were different in the four TB varieties. Under HN treatment, the expression of *FtZOX1* (FtPinG0009420300.01) in YQ1, YQ2, and XQ2 was up-regulated, and the expression in FH was down-regulated. The expression of *FtPP2C28* (FtPinG0005550700.01) was down-regulated in YQ1, and HN treatment significantly up-regulated *FtPP2C28* expression in YQ2, XQ2, and FH (**Figure 5B**).

Ten DEGs involved in N transport and assimilation were found in the 426 common NH_4_^+^-responsive DEGs (**Supplementary Table 5**). Most of these genes’ expression involved in N transport was significantly inhibited by HN treatment, but HN treatment significantly induced the expression of *FtNRT1.10* (FtPinG0004671100.01) (**Figure 5C**). The two genes involved in N assimilation, *FtNIR* and *FtGDH1*, showed different expression patterns. HN treatment significantly induced the expression of *FtGDH1* (FtPinG0008184600.01), while the expression of *FtNIR* (FtPinG0006174600.01) was significantly inhibited by HN (**Figure 5C**).

**Figure 6:**
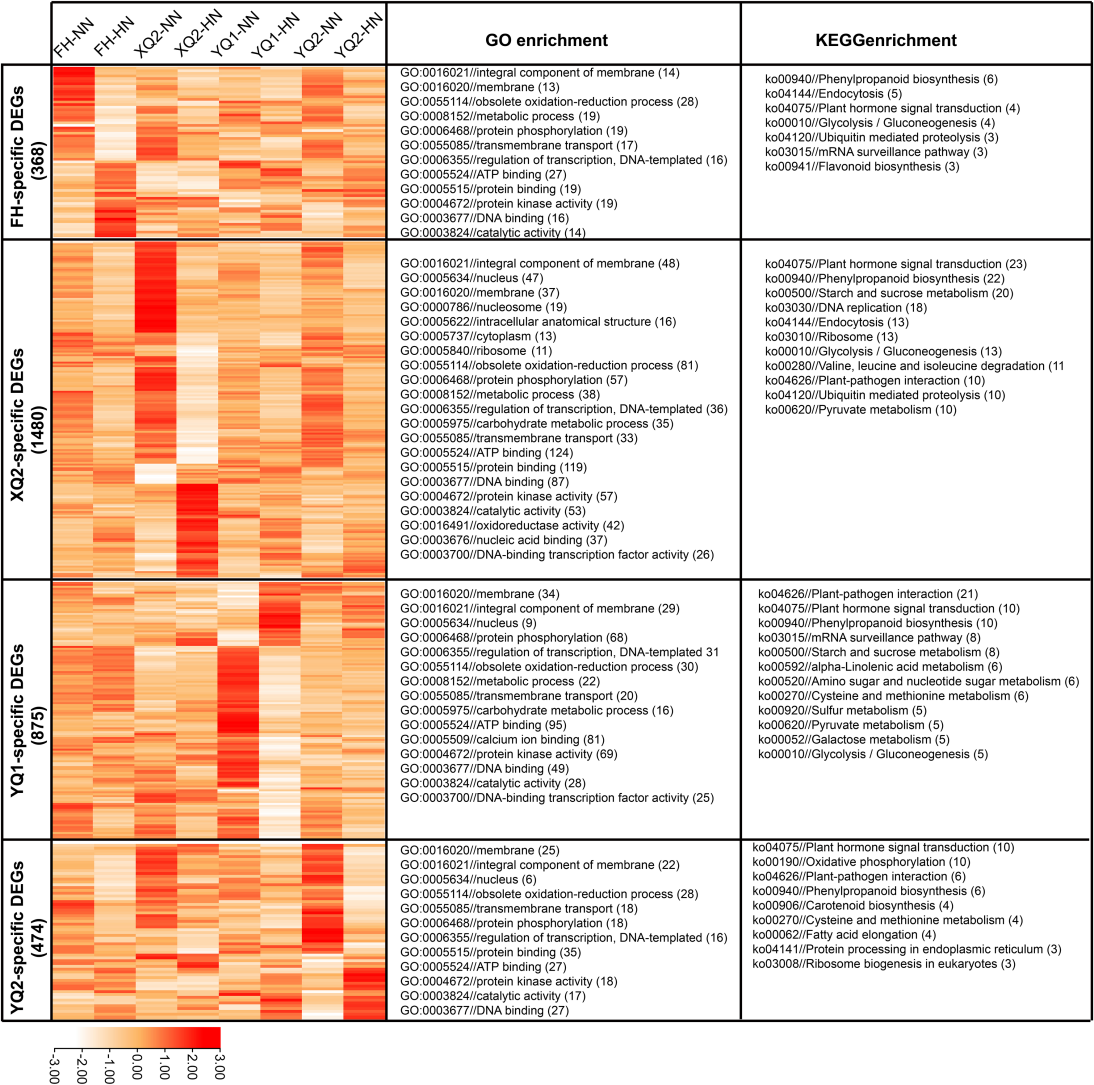
Expression analysis and GO/KEGG enrichment of the genotype-specific high NH_4_^+^-responsive DEGs. Color scale in the heatmaps indicates the degree of expression: red, high expression; white, low expression.

In this study, 28 TFs were identified from the 426 common DEGs (**Supplementary Table 5**). Within *MYB* genes, the expression of four genes (*FtMYB39/52/80* and *FtECT1*) was significantly up-regulated by HN, and two genes (*FtMYB61/83*) was inhibited by HN (**Figure 6D**). In NAM, ATAF1/2 and CUC2 protein (NAC) genes, there were four up-regulated genes (*FtNAC10/29/42/72*) and one down-regulated gene (*FtNAC73*). In *bZIPs*, there were two up-regulated genes (*FtbZIP1/53* and *FtHY5*) and two down-regulated genes (*FtbZIP6/34*). Within *WRKYs*, there were two up-regulated genes (*FtWRKY61/75*) and one down-regulated gene (*FtWRKY41*). Within homeobox-leucine zipper proteins (HD-ZIP) and *MADS* genes, *FtHAT22* and *FtMADS14* are up-regulated DEGs and *FtHDG1/FtAGL8* are down-regulated DEGs (**Figure 5D**). In addition, HN treatment significantly induced the expression of *FtLBD38* and *FtHY5*, and significantly inhibited the expression of *FtPIF3*, *FtNF-YA10*, *FtANT*, and *FtbHLH62* (**Figure 5D**).

In our previous study, 932 genes encoding small secreted peptides (SSPs) were reported (Liu et al., 2021b). In this paper, 19 high NH_4_^+^-responsive SSPs encoding genes were identified by Venn diagram analysis. Seven of these SSPs was identified as established SSPs, including one CLAVATA3/EMBRYO SURROUNDING REGION proteins (CLE), one C-terminally encoded peptide (CEP), two germin-like proteins (GLPs), two lipid transfer proteins (LTPs), and two plant defensin (PDF). Expression analysis showed that the 19 *SSP* genes performed different expression patterns in response to high NH_4_^+^ (**Figure 5E**; **Supplementary Table 5**). The expression of eight *SSP* genes including *FtCEP3*, *FtGLP1*, and *FtLTP14* was down-regulated by HN, while ten genes including *FtCLE7*, *FtPDF8/14*, and *FtGLP31* showed up-regulated expression pattern in all four varieties. Besides, the expression *FtLTP7* was down-regulated by HN in XQ, while HN enhanced its expression level in other three varieties (**Figure 5F**).

In addition, the correlations among the key high NH_4_^+^-responsive DEGs were analyzed, including DEGs involved in antioxidant enzyme system, hormone signaling, and N transport/assimilation, as well as TFs and SSPs encoding genes (**Figure 5G**). 70 of 75 these genes showed high correlation with the expression of one or more genes. Some genes, such as *FtPER60-1*, *FtASOL-1*, *FtACS7*, *FtACO1/5*, *FtNRT1.14*, *FtMYB61/52*, *FtANT*, *FtbZIP6/34*, *FtNAC72/73*, *FtLTP14*, and *FtCEP3*, may be the core genes in regulating high NH_4_^+^ response of TB (**Figure 5G**).

### 3.5 Identification of genotype-specific high NH_4_^+^-responsive DEGs

In our previous studies, the four varieties, XQ2, FH, YQ1, and YQ2, showed different sensitive to N in hydroponic and field environments, and the genotype-specific N-responsive genes was revealed (Qiu et al., 2023; Liu et al., 2023a). Therefore, these four varieties were usually used for investigating the physiological mechanism of TB in response to N in our research, including high NH_4_^+^. In this study, the four varieties were clustered based the fold changes of root weight, total root length, primary root length, root surface area, and root volume under 1 and 100 mmol/L NH_4_^+^. The four varieties showed different sensitivity to HN, and FH is less sensitive to HN stress than other three varieties (**Supplementary Figure 3**). For this, the difference of four varieties in response to HN at transcriptome level was investigated. 368, 1,480, 875, and 474 specific DEGs were found in FH, XQ2, YQ1, and YQ2, respectively (**Figure 6**). GO enrichment analysis showed that the 368 FH-specific DEGs were more enriched into integral component of membrane, membrane, obsolete oxidation-reduction process, metabolic process, protein phosphorylation, regulation of transcription, DNA-templated, protein kinase activity, and DNA binding. 1, 480 XQ2-specific DEGs were more enriched into integral component of membrane, nucleus, obsolete oxidation-reduction process, protein phosphorylation, metabolic process, regulation of transcription, DNA-templated, ATP binding, protein binding, and DNA binding. 875 YQ1-specific DEGs were more enriched into membrane, integral component of membrane, protein phosphorylation, regulation of transcription, DNA-templated, obsolete oxidation-reduction process, calcium ion binding, protein kinase activity, and DNA binding. 474 YQ1-specific DEGs were more enriched into membrane, integral component of membrane, nucleus, obsolete oxidation-reduction process, transmembrane transport, protein phosphorylation, regulation of transcription, DNA-templated, protein binding, ATP binding, and protein kinase activity (**Figure 6**).

KEGG enrichment analysis showed that the 368 FH-specific DEGs were more enriched into phenylpropanoid biosynthesis, endocytosis, plant hormone signal transduction, glycolysis, and ubiquitin mediated proteolysis. 1, 480 XQ2-specific DEGs were more enriched into plant hormone signal transduction, phenylpropanoid biosynthesis, starch and sucrose metabolism, DNA replication, endocytosis, and ribosome. 875 YQ1-specific DEGs were more enriched into plant-pathogen interaction, plant hormone signal transduction, phenylpropanoid biosynthesis, mRNA surveillance pathway and starch, and sucrose metabolism. 474 YQ1-specific DEGs were more enriched into plant hormone signal transduction, oxidative phosphorylation, plant-pathogen interaction, phenylpropanoid biosynthesis, and carotenoid biosynthesis (**Figure 6**).

### 3.6 Integrating genomic and transcriptome analysis to identify key high NH_4_^+^-responsive DEGs

**Figure 7:**
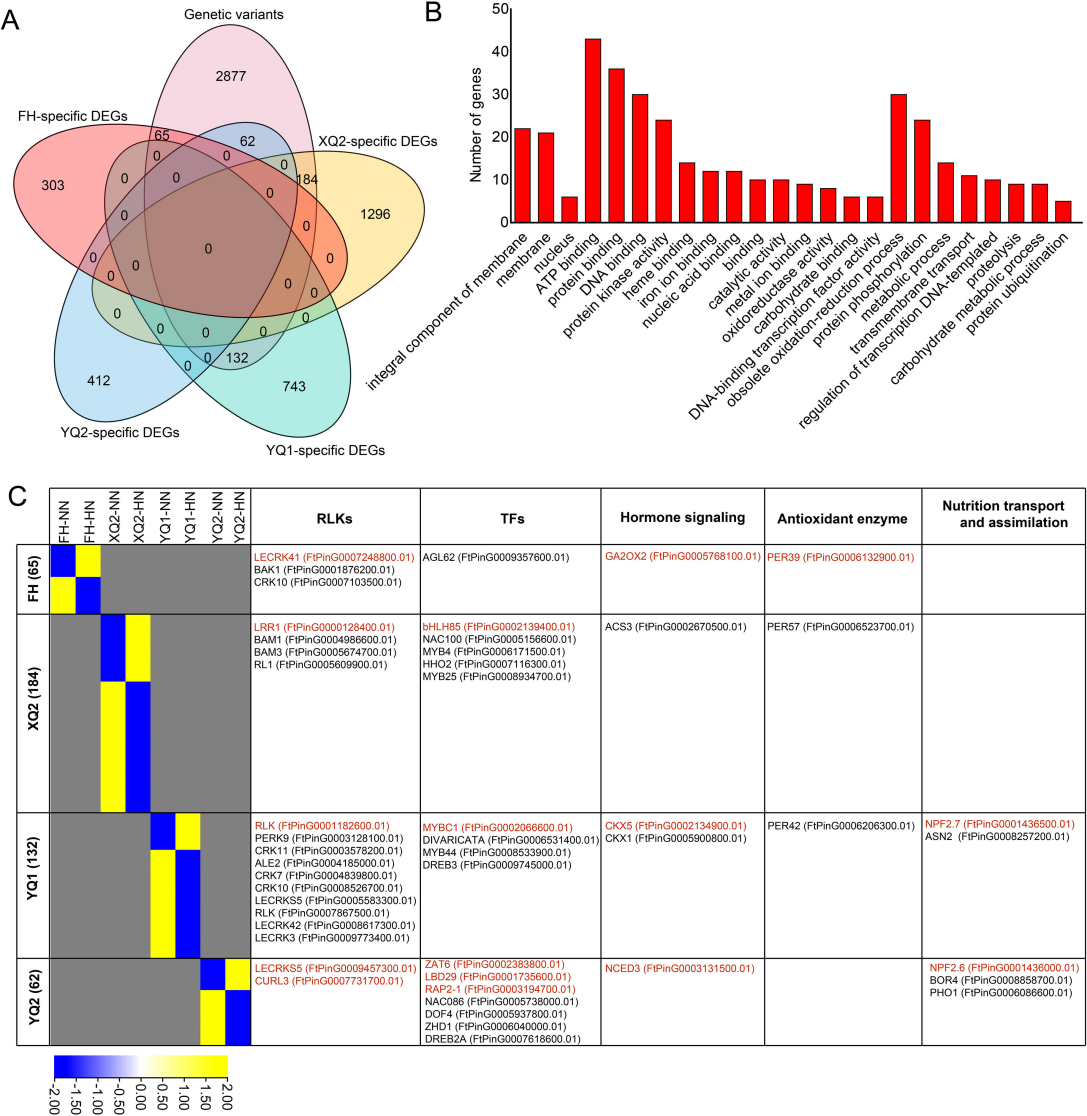
Identification of the key genotype-specific high NH_4_^+^-responsive DEGs with sequence variation. **(A)** Venn diagram showing the genotype-specific varied DEGs. **(B)** GO enrichment analysis of the 443 genotype-specific varied DEGs. **(C)** Heatmap analysis of the 443 genotype-specific varied DEGs. Color scale in the heatmaps indicates the degree of expression: yellow, high expression; blue, low expression. The genes marked red fond represent the up-regulated genes under high NH_4_^+^. The genes marked black fond represent the down-regulated genes under high NH_4_^+^.

In a previous study, FH, XQ2, YQ1, and YQ2 were analyzed by genomic re-sequencing and generating 3,220 varied genes (Qiu et al., 2023). 443 genotype-specific high NH_4_^+^-responsive DEGs with genome sequence variation were found, including 65 FH-specific varied DEGs, 184 XQ2-specific varied DEGs, 132 YQ1-specific varied DEGs, and 62 YQ2-specific varied DEGs (**Figure 7A**). GO enrichment analysis showed that the 443 genotype-specific varied DEGs were more enriched into integral component of membrane, membrane, ATP binding, protein binding, DNA binding, protein kinase activity, heme binding, obsolete oxidation-reduction process, protein phosphorylation, metabolic process, transmembrane transport, and regulation of transcription DNA-templated (**Figure 7B**).

The key **h**igh NH_4_^+^-responsive varied DEGs involved in several pathways were analyzed (**Figure 7C**). In FH, three receptor-like protein kinases (*RLK*), one TF, one hormone signaling, and one *PER* genes were found. Four *RLK* (such as *BAM1/3*), five *TF* (such as *MYB4/25*), one hormone signaling, and one *PER* genes were found in XQ2. In YQ1, ten *RLK* (such as *LECRK3/5/42* and *CRK7/10/11*), one TF (such as *MYB44/C1*), two hormone signaling, one *PER*, and two N transport and assimilation-related gene were found. In addition, two RLK, seven TF (such as *LBD29* and *RAP2-1*), one hormone signaling, and N transport and assimilation-related genes were found in YQ2 (**Figure 7C**). Above data provide the first-hand data for revealing the mechanism of TB root respond to high NH_4_^+^.

## 4 Discussion

### 4.1 High NH_4_^+^ inhibits root growth by affecting the antioxidant enzyme system

N is one of the key limiting factors for plant growth and crop yield (Teng et al., 2022). Previous studies have shown that an appropriate amount of N fertilizer can significantly promote plant growth, flowering, fruiting, and yield, but high NH_4_^+^ can inhibit plant growth (Hikosaka, 2004; Wang et al., 2022a). Improving the knowledge on physiological and molecular mechanisms of plants respond to high NH_4_^+^ will contribute to the improvement of the crops with high NH_4_^+^ tolerance and high N use efficiency. In this study, it is found that high NH_4_^+^ affected TB growth by hydroponic experiment, and the growth of TB root was severely limited (**Figure 1**). It is speculated that high NH_4_^+^ significantly affects the growth of roots and causes its toxicity, which in turn affects shoots growth (Liu et al., 2023b). In order to investigate the effect of high NH_4_^+^ on TB root, the physiological and gene expression changes involved in antioxidant enzyme system were analyzed by biochemical and transcriptome analyses. High NH_4_^+^ significantly increased the MDA content of TB plants and affected the activities of CAT, POD, APX, and SOD (**Figure 2**). In tomato (*Solanum lycopersicum* L.), the activities of antioxidant enzymes including SOD, POD, and CAT were also affected significantly by high NH_4_^+^ (Zhang et al., 2022). Most of the genes encoding POD and APX were down-regulated by high NH_4_^+^ (**Figure 5A**). The above analysis indicated that high NH_4_^+^ inhibits root growth by affecting the antioxidant enzyme system, thereby suppressing plant growth. Previous studies showed that graphene oxide nanomaterials and cadmium stress enhanced CAT activity while decreased POD activity in TB roots, which is completely opposite to the results of this study (Liu et al., 2022; Ye et al., 2022). Under drought stress, SOD, CAT, and POD activities in the root of TB and its close relative species, common buckwheat (*Fagopyrum esculentum* Moench) and *Fagopyrum leptopodum*, were up-regulated (Wang et al., 2023a, 2025; Zhu et al., 2022a). Aluminum toxicity enhanced CAT activity but did not affect SOD activity in common buckwheat roots (Salazar-Chavarría et al., 2020). However, NaCl stress suppressed the activities of CAT, POD, and APX in TB roots (Yao et al., 2022). The above analysis indicates that TB roots showed diverse and complex responses to different abiotic stresses at biochemical level, the underlying mechanism is worth to be explored deeply in future study.

### 4.2 426 common DEGs suggest the mechanism on *Fagopyrum* genus respond to high NH_4_^+^

In recent years, many works for studying the mechanisms of TB in response to N were conducted, and TB was a potential model to research N response in plants (Liu et al., 2021a, 2021b, 2023a; Qiu et al., 2023). However, the mechanism on TB responds high NH_4_^+^ stress remains unclear. In this study, 426 common high NH_4_^+^-responsive DEGs were found in all four TB varieties (**Figure 4B**). It is found that the expression of genes involved in N uptake and utilization was significantly inhibited by high NH_4_^+^ (**Figure 5C**; **Supplementary Table 5**). These results are similar to the data that was reported in a previous study (Sun et al., 2021b). It is indicated that high NH_4_^+^ affects nutrient uptake by inhibiting the expression of nutrient uptake and utilization genes. Here, a key N transport gene, *FtNRT1.14*, showed decreased expression under high NH_4_^+^ condition (**Figure 5C**). Previous studies showed that *NRT1.1* reduced ammonium tolerance of *Arabidopsis*, and mutation of *NRT1.1* enhanced high NH_4_^+^ resistance (Xiao et al., 2022). The down-regulation of *FtNRT1.14* expression may help TB to survive from high NH_4_^+^. In addition, the expression of *FtGDH1* encoding glutamate dehydrogenase was significantly inhibited by high NH_4_^+^ (**Figure 5C**). In rice, overexpression of a fungal *GDH* gene improved tolerance to ammonium toxicity by increasing assimilation of excess NH_4_^+^ (Yan et al., 2021), which indicated that manipulating the ammonium assimilation may be a promising strategy for improving high NH_4_^+^ tolerance of crops.

Previous studies suggested that hormones regulated ammonium response in plants (Li et al., 2014, 2019; Sun et al., 2020; Pandey et al., 2024). In this study, the ETH biosynthetic genes, *FtACO1/5* and *FtACS1/7*, showed decreased expression under high NH_4_^+^ treatment (**Figure 5B**). Thus, ETH accumulation and its signaling in root may be suppressed by high NH_4_^+^. Previous studies showed that high NH_4_^+^ enhanced ethylene biosynthesis in shoots by up-regulating *ACO* and *ACS* genes, and then leading to oxidative stress under NH_4_^+^ stress (Li et al., 2014, 2019). Meanwhile, mutation of ETH signaling components such as ETR1 and EIN3 show greater NH_4_^+^ tolerance of *Arabidopsis*, while mutation of these negative regulators of ethylene biosynthesis improved sensitivity to high NH_4_^+^ (Li et al., 2013, 2014). Above analysis indicated that ETH showed different response in root and shoot, and the suppression of ETH signaling in root may be better for TB to adapt to high NH_4_^+^ environment.

SSP is a kind of mature polypeptide with special functions formed by proteasome cleavage, and its length is usually 5~20 amino acids (Boschiero et al., 2020). In recent years, many studies have shown that plant SSPs play an important role in N uptake and utilization. CEP and CLE peptides are main SSPs involved in the regulation of N signal transduction. CEP and CLE promote N uptake and utilization in *Arabidopsis*, soybean (*Glycine max* L.), *Lotus japonicus*, and alfalfa (*Medicago truncatula*) (Laffont et al., 2020; Lim et al., 2011; Okamoto et al., 2013, 2016). In this study, a *CLE* gene, *FtCLE7*, and a *CEP* gene, *FtCEP3*, were found in the 426 high NH_4_^+^-responsive genes (**Figure 5F**). The expression of *FtCLE7* was improved by high NH_4_^+^, while *FtCEP3* expression was inhibited (**Figure 5F**). These two genes showed higher correlations with genes involved in antioxidant enzyme system, hormone signaling, and transcriptional regulation (**Figure 5G**). These data indicated that *FtCLE7* and *FtCEP3* may be the core genes in high NH_4_^+^ response. In a previous study in alfalfa, MtCLE13 and MtCEP7 played antagonistic roles in rhizobial infection, nodulation, and N signaling (Laffont et al., 2020). It is suggested that the up-regulation of *FtCLE7* expression and down-regulation of *FtCEP3* expression may represent a finely tuned balancing mechanism, aiming to optimize root perception and response to N in a high NH_4_^+^ environment. However, more studies are needed to clarify this hypothesis.

In previous studies, the functions of many TFs in regulating various abiotic and biotic stresses have been widely studied in TB. Previous studies showed found that FtNAC31 enhances salt and drought tolerance in transgenic *Arabidopsis* by increasing antioxidant enzyme activities and stress-associated genes’ expression, and FtNAC10 enhanced root elongation under saline and drought stress through ABA-signaling pathway (Li et al., 2023; Zhao et al., 2022). *FtNAC10/29/42/72* genes, especially the hub gene *FtNAC72*, were found to be up-regulated by high NH_4_^+^ (**Figures 5D, G**), which may be the broad-spectrum resistance gene against abiotic stresses in TB. Some MYB in TB such as FtMYB9/13/30 regulated salt/drought tolerance by regulating different stress-responsive signaling pathways, and FtMYB12 improves cold tolerance (Gao et al., 2017; Wang et al., 2022b; Huang et al., 2018; Zhou et al., 2015). FeR2R3-MYB in common buckwheat positively regulates anthocyanin biosynthesis and drought tolerance (Luo et al., 2024). In this study, many high NH_4_^+^-responsive *MYB* genes including *FtMYB38/52/80* were found, these genes may mediate the crosstalk among different abiotic stresses (**Figure 5D**). In addition, three WRKY encoding genes, *FtWRKY41/61/75*, were found to be regulated by high NH_4_^+^ (**Figure 5D**). It is known that the WRKY widely regulating plant immunity and pathogen response. It was found that FtWRKY29 improved the tolerance to low phosphorus stress in TB, and FtWRKY46 improves salt tolerance by regulating the expression of stress-related gene (Lv et al., 2020; Wang et al., 2023b). Therefore, WRKY TFs may be the important regulatory factors for improving the tolerance and adaptation of TB to varied stress environments.

### 4.3 Transcriptome and genomic analysis revealed the potential genes for improving the tolerance of TB to high NH_4_^+^

In this study, 443 genotype-specific high NH_4_^+^-responsive DEGs with genome sequence variation were found (**Figure 7**). FH showed less sensitive to HN stress than other three varieties (**Supplementary Figure 3**), these NH_4_^+^-responsive genes including *FtPER39*, *FtGA2OX2*, and *FtAGL62* may help FH to cope with high NH_4_^+^ stress (**Figure 7C**). *FtPER39* (FtPinG0002970300.01) encodes peroxidase, which can hydrolyze hydrogen peroxide and protect plants from stress. The expression of *FtGA2OX2* (FtPinG0000400800.01) encoding gibberellin 2-beta-dioxygenase, the key enzyme in GA signaling, was up-regulated by high NH_4_^+^. *OsGA2ox8* in rice improved the tolerance of plants to osmotic stress (Wang et al., 2021), and *Arabidopsis AtGA2ox9* contributes to freezing tolerance (Lange et al., 2020). In a recent study, *Arabidopsis GA2OX6* and *GA2OX8* regulated root cell elongation by mediating IAA-GA cross talk (Kubalová et al., 2025). Thus, it is indicated that the up-regulation of *FtGA2OX2* may contribute to high NH_4_^+^ tolerance by regulating root growth. The *MADS* gene *FtAGL62* showed significant response to high NH_4_^+^ in FH. A *MADS* gene in barley, *HvMADS27*, regulates root architecture in response to high NH_4_^+^ (Smoczynska et al., 2022). Meanwhile, many other TFs were found from the 443 genotype-specific DEGs in other three varieties, such as *MYB* and *LBD* genes (**Figure 7C**). In a previous study suggested *MYB28* and *MYB29* improved the tolerance of *Arabidopsis* to high NH_4_^+^ (Coleto et al., 2021). High NH_4_^+^ treatment significantly induced the expression of *FtLBD29* in TB (**Figure 7C**). In rice, OsLBD37/38/39 collaboratively alleviate excessive N stress by inhibit the expression of *OsNRT2.1/2.2/2.3* (Zhu et al., 2022b). These TFs provided candidate genes for investigating the transcriptional regulation mechanism of TB respond to high NH_4_^+^.

There are 19 genes encoding RLK were found in the 443 genotype-specific DEGs, and most of these genes were inhibited by high NH_4_^+^ (**Figure 7C**). Two *RLK* genes, BAM1 (FtPinG0004986600.01) and BAM3 (FtPinG0005674700.01) encoding BARELY ANY MERISTEM. In *Arabidopsis*, BAM1/2/3 were identified as the receptor of CLE peptide mediated signaling, and the participating in regulating root meristem tissue patterning and protophloem differentiation driven by CLE (Hu et al., 2022; Zhang et al., 2024). In this study, a *CLE* gene, *FtCLE7*, showed response to high NH_4_^+^ (**Figure 5F**), which indicated that it may be worthy to research the function of FtCLE7-BAM1/3 signaling in high NH_4_^+^ response. In addition, SSP acts as a signal molecule to exchange information between cells and cells by interacting with RLKs on the cell membrane, thereby activating downstream genes or initiating related signal transduction processes (Matsubayashi, 2014). Thus, these genotype-specific varied *RLK* genes provide the candidates for identifying the receptor of SSP in high NH_4_^+^ response.

### 4.4 The proposed working model for TB in response to high NH_4_^+^ was suggested

**Figure 8:**
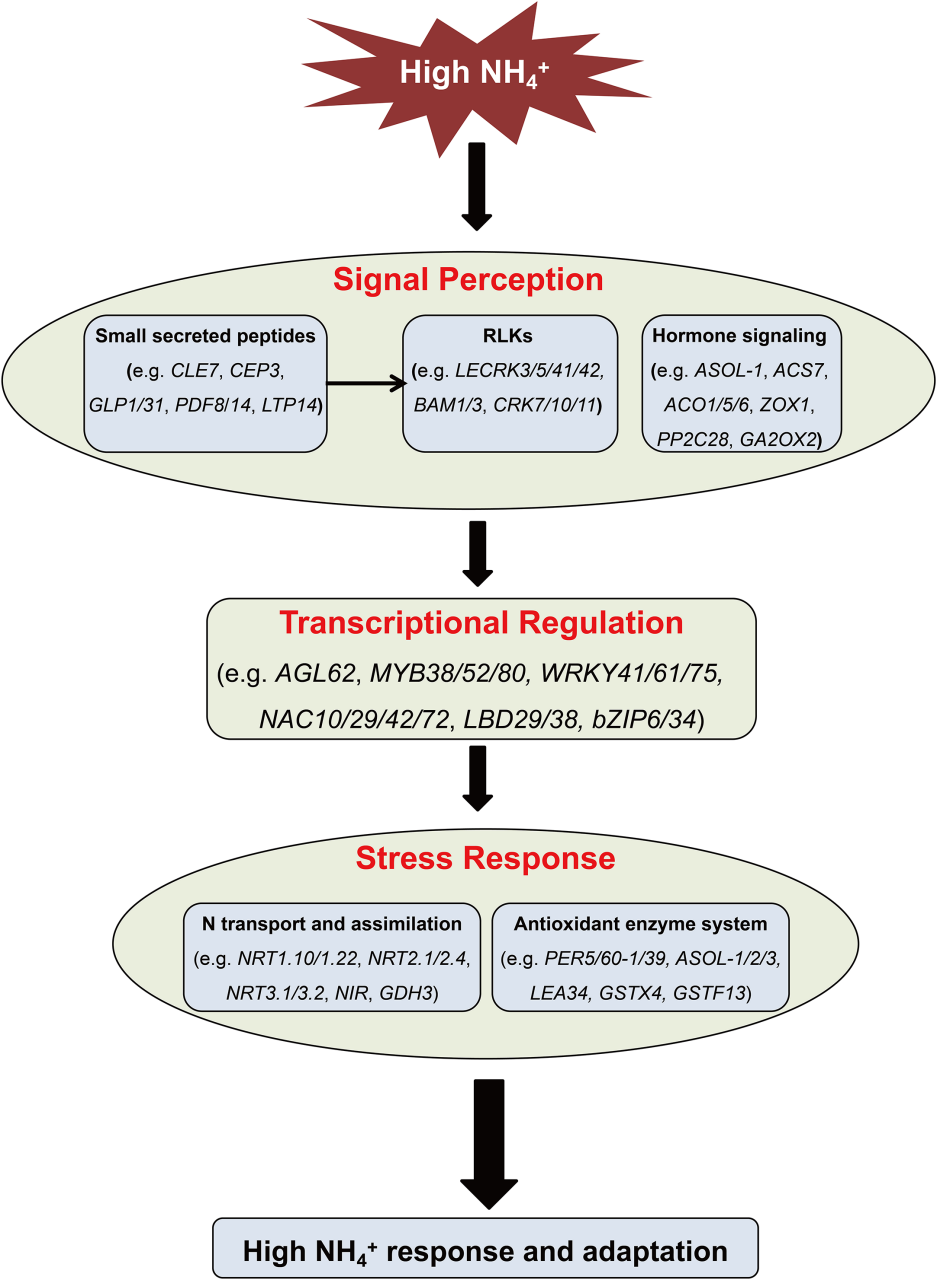
The proposed working model for TB in response to high ammonium stress.

Based on the above analysis, the working model for TB in response to high NH_4_^+^ is summarized in **Figure 8**. High NH_4_^+^ stress is initially perceived by RLKs, SSP, and hormone-mediated signaling in root cells, and then transmitting signals to TFs. These TFs then regulate the expression of downstream genes. This might involve inhibiting most N transport and assimilation genes to mitigate toxicity, and modulating the antioxidant enzyme system to scavenge reactive oxygen species. In this model, the key SSP, FtCLE7 and FtCEP3 may pass the stress signals to their receptors belonging to RLKs on cell membrane. Meanwhile, down-regulation of ETH biosynthesis and signaling may help TB to adapt to high NH_4_^+^ environment by maintaining root growth.

## 5 Conclusion

In conclusion, the morphological and transcriptome changes of roots in TB under high NH_4_^+^ conditions were analyzed for the first time. High NH_4_^+^ reduced TB growth through hydroponic experiment. High NH_4_^+^ inhibits root growth and causes its toxicity, which in turn affects shoots growth. Biochemical analysis showed that high NH_4_^+^ resulted in the changes in the activities of antioxidant enzymes in roots. The roots of the TB seedlings under high NH_4_^+^ conditions were selected for transcriptome analysis. In total 426 High NH_4_^+^-responsive DEGs were identified. Most of the DEGs involved in antioxidant enzyme system, hormone biosynthesis and signaling, and N transport and assimilation were down-regulated by high NH_4_^+^. Suppression of ETH signaling in root may be better for TB to adapt to high NH_4_^+^ environment. 19 SSPs encoding genes were found to be involved in high NH_4_^+^ response, including *FtCLE7* and *FtCEP3*. The up-regulation of *FtCLE7* expression and down-regulation of *FtCEP3* expression may help plants to optimize root perception and response to high NH_4_^+^. The core genes in regulating high NH_4_^+^ response of TB were identified by co-expression analysis, such as *FtNRT1.14*, *FtMYB61/52*, *FtbZIP6/34*, *FtNAC72/73*, *FtLTP14*, and *FtCEP3*. Additionally, 443 genotype-specific high NH_4_^+^-responsive DEGs with sequence variation were identified by integrating transcriptome and whole-genome re-sequencing analysis, including 17 *TFs* such as *MYB*, *MADS*, and *LBD* and 19 *RLKs* such as *FtBAM1/3*. This study will provide new insight into molecular response to high NH_4_^+^, and provides candidate genes for improving N use efficiency and high NH_4_^+^ tolerance of crops through genetic manipulation.

## Data Availability

The datasets presented in this study can be found in online repositories. The names of the repository/repositories and accession number(s) can be found in the article/supplementary material.

## Supplementary material

Supplementary Figure 1 Architectural changes of seedlings as affected by high NH_4_^+^.

Supplementary Figure 2 Validation of expression profile through qRT-PCR. A total of 12 genes were validated using qRT-PCR against their expression profile from RNA-seq. The relative expression levels of gene were quantitated using 2^–ΔΔCt^.

Supplementary Figure 3 Cluster analysis of four TB varieties based the fold changes of root weight, total root length, primary root length, root surface area and root volume under 1 and 100 mmol/L NH_4_^+^.
