# Supplementary figures and images for: Physiological, transcriptomic, and genomic analysis unravels the response of Tatary buckwheat root to high ammonium stress
Source: Front Plant Sci. 2025 Oct 27;16:1669365. doi: 10.3389/fpls.2025.1669365 (PMC12597952; doi:10.3389/fpls.2025.1669365)

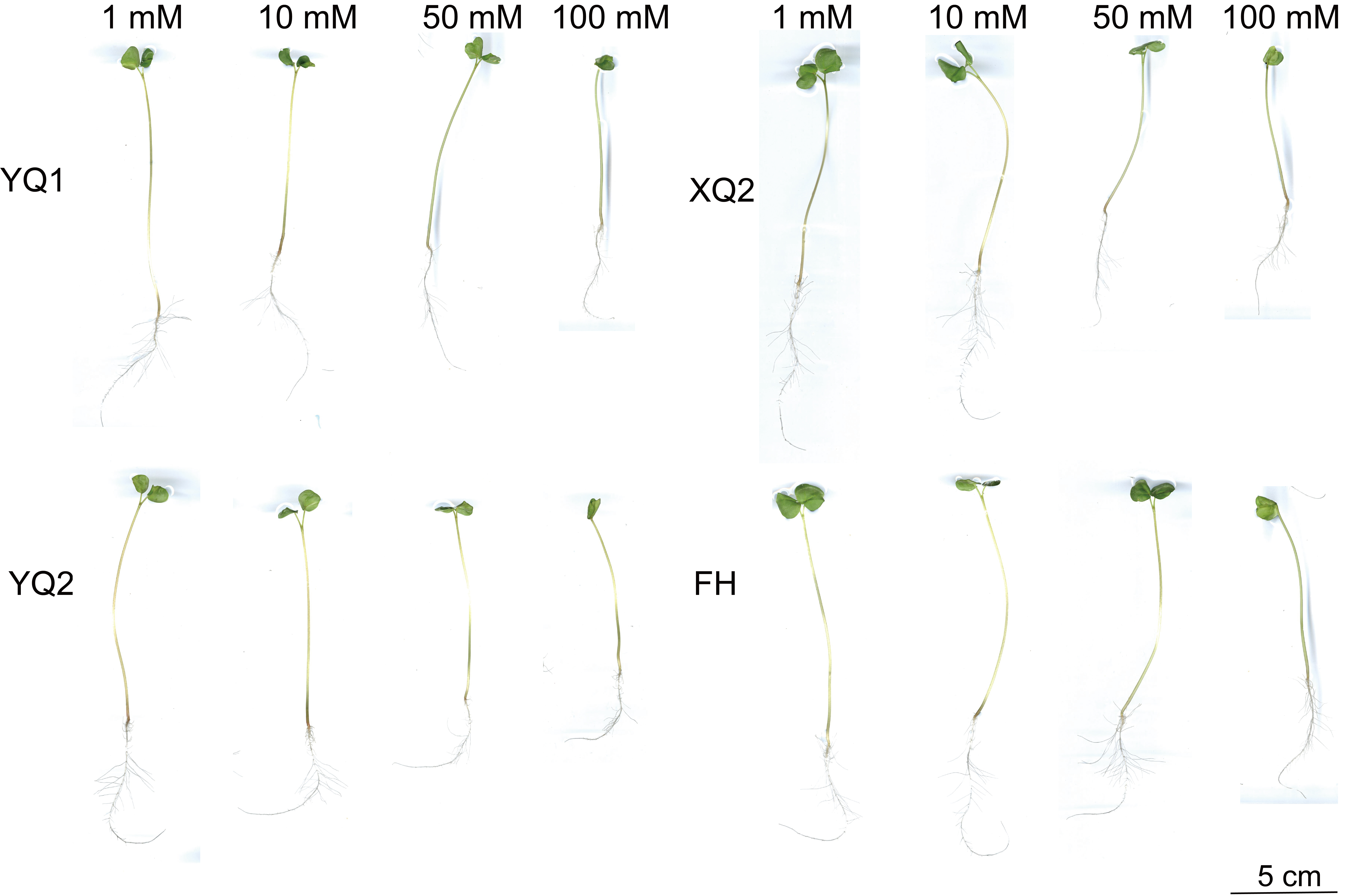

Supplement: Supplementary Figure 1 — Architectural changes of seedlings as affected by high NH4 +. [file Image1.jpeg]

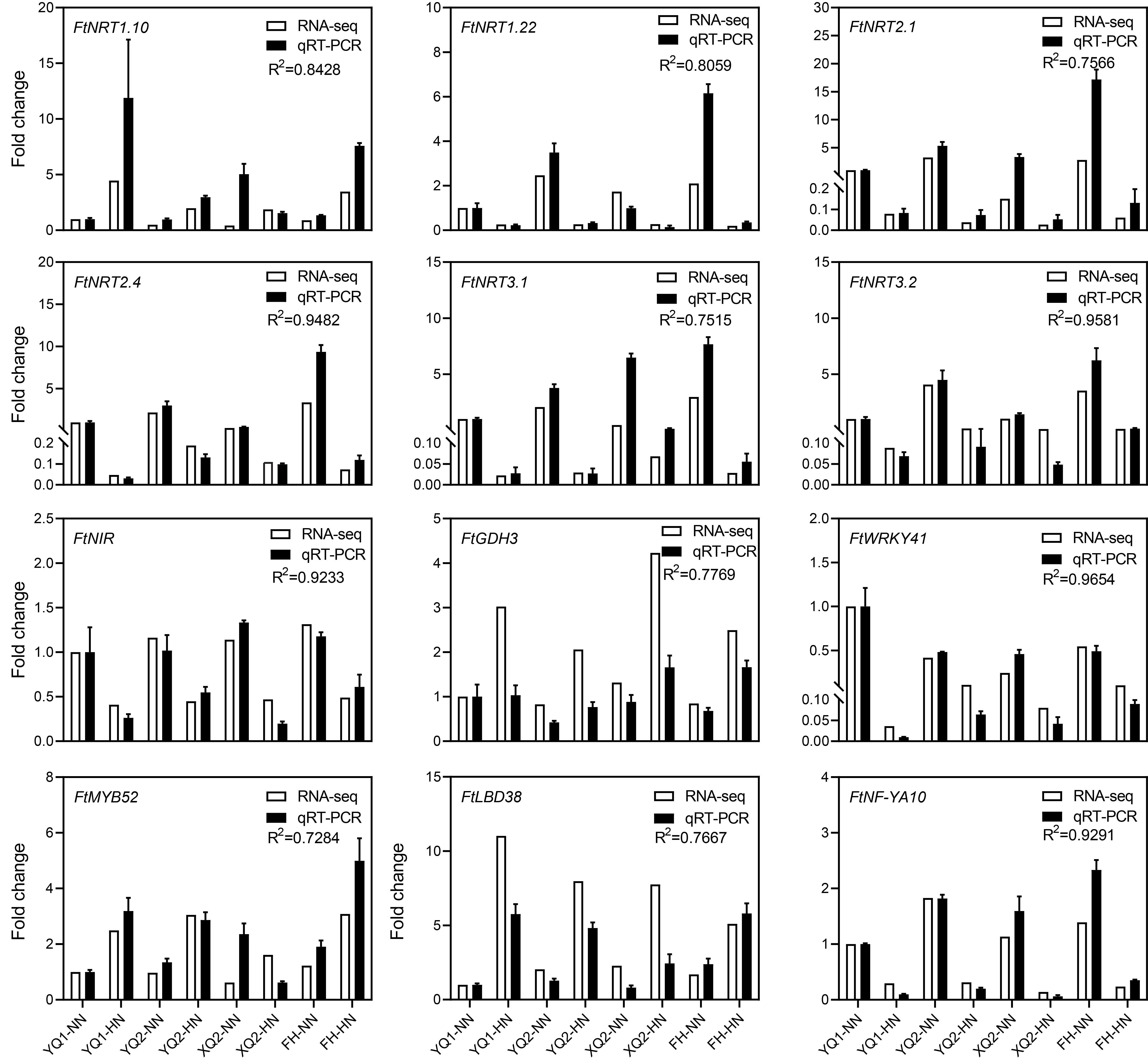

Supplement: Supplementary Figure 2 — Validation of expression profile through qRT-PCR. A total of 12 genes were validated using qRT-PCR against their expression profile from RNA-seq. The relative expression levels of gene were quantitated using 2–ΔΔCt. [file Image2.jpeg]

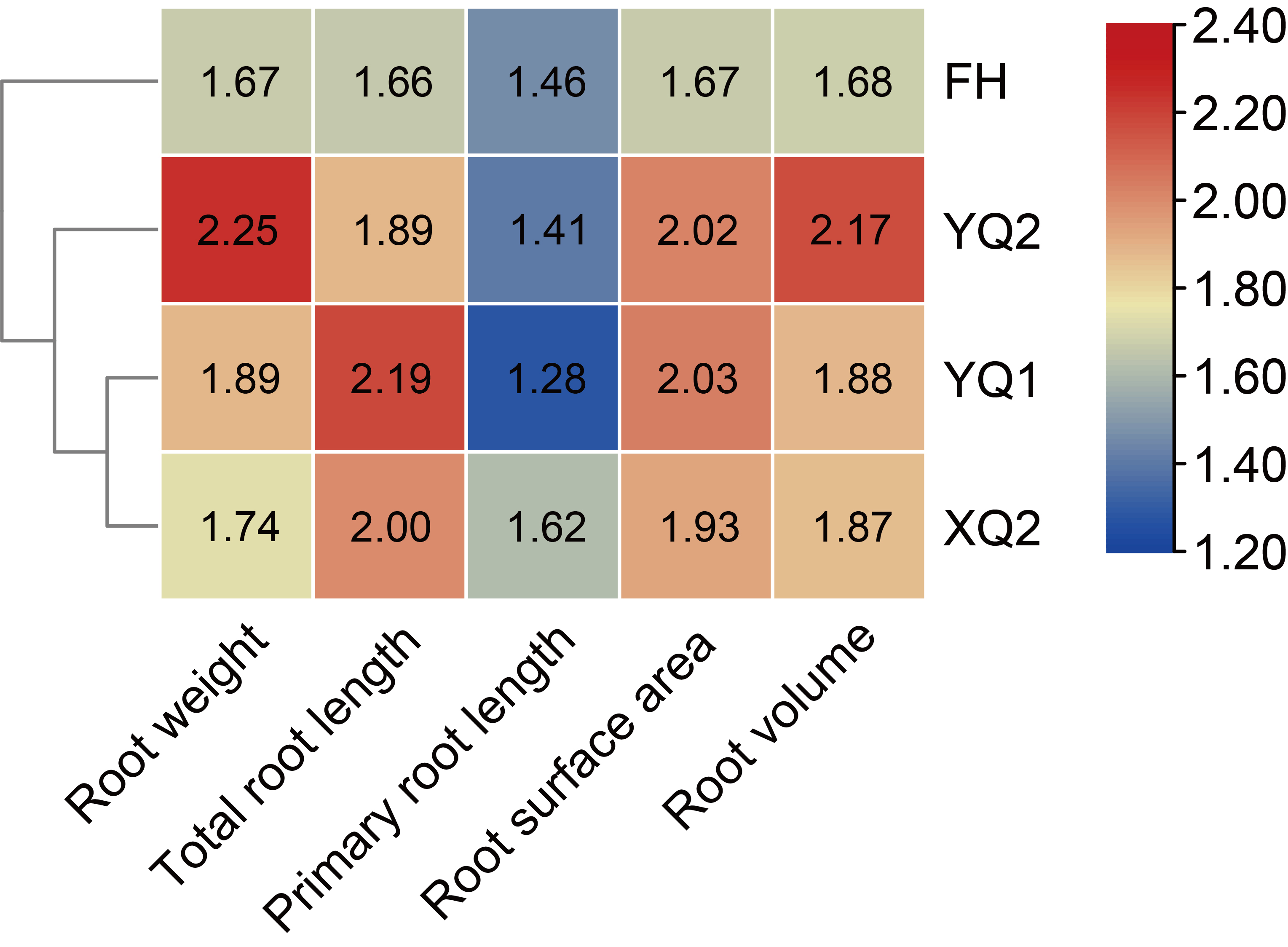

Supplement: Supplementary Figure 3 — Cluster analysis of four TB varieties based the fold changes of root weight, total root length, primary root length, root surface area and root volume under 1 and 100 mmol/L NH4 +. [file Image3.jpeg]
